# Identification of Novel Equine (*Equus caballus*) Tendon Markers Using RNA Sequencing

**DOI:** 10.3390/genes7110097

**Published:** 2016-11-10

**Authors:** Jan M. Kuemmerle, Felix Theiss, Michal J. Okoniewski, Fabienne A. Weber, Sonja Hemmi, Ali Mirsaidi, Peter J. Richards, Paolo Cinelli

**Affiliations:** 1 Center for Applied Biotechnology and Molecular Medicine, University of Zurich, Winterthurerstrasse 190, CH-8057 Zurich, Switzerland; jkuemmerle@vetclinics.uzh.ch (J.M.K.); ftheiss@vetclinics.uzh.ch (F.T.); ali.mirsaidi@cabmm.uzh.ch (A.M.); 2 Equine Hospital, Vetsuisse Faculty, University of Zurich, CH-8057 Zurich, Switzerland; 3 Scientific IT Services, Swiss Federal Institute of Technology, CH 8092 Zurich, Switzerland; michalo@id.ethz.ch; 4 Institute of Laboratory Animal Science, University of Zurich, CH-8057 Zurich, Switzerland; fabienne.weber@lasc.uzh.ch; 5 Division of Trauma Surgery, Center for Clinical Research, University Hospital Zurich, University of Zurich, Sternwartstrasse 14, CH-8091 Zurich, Switzerland; Sonja.hemmi@usz.ch

**Keywords:** horse, tendon, transcriptome, eyes absent homolog 2, G-protein regulated inducer of neurite outgrowth 3

## Abstract

Although several tendon-selective genes exist, they are also expressed in other musculoskeletal tissues. As cell and tissue engineering is reliant on specific molecular markers to discriminate between cell types, tendon-specific genes need to be identified. In order to accomplish this, we have used RNA sequencing (RNA-seq) to compare gene expression between tendon, bone, cartilage and ligament from horses. We identified several tendon-selective gene markers, and established eyes absent homolog 2 (*EYA2*) and a G-protein regulated inducer of neurite outgrowth 3 (*GPRIN3*) as specific tendon markers using RT-qPCR. Equine tendon cells cultured as three-dimensional spheroids expressed significantly greater levels of *EYA2* than *GPRIN3*, and stained positively for EYA2 using immunohistochemistry. EYA2 was also found in fibroblast-like cells within the tendon tissue matrix and in cells localized to the vascular endothelium. In summary, we have identified *EYA2* and *GPRIN3* as specific molecular markers of equine tendon as compared to bone, cartilage and ligament, and provide evidence for the use of EYA2 as an additional marker for tendon cells in vitro.

## 1. Introduction

Tendinopathy is one of the most frequently diagnosed sports-related injury both in human and equine athletes [1,2,3], predominantly affecting energy-storing tendons such as the superficial digital flexor tendon (SDFT) in horses [3] and the Achilles tendon in humans [4]. The failure to achieve regeneration of injured tendon tissue is thought to be the result of an inadequate intrinsic cellular healing response [5,6]. The regenerative approach to tendon healing aims to regenerate tendon tissue through the application of growth factors or implantation of stem cells [5,7,8,9,10,11]. Indeed, the clinical use of mesenchymal stem cells (MSCs) to treat horses suffering from tendon injury was introduced over 10 years ago [12]. Furthermore, several in vitro and in vivo studies have since been conducted to evaluate various aspects of stem cell therapy of tendon injury in the horse [13,14,15,16]. Since it is known that the pathophysiology of tendon injury and the healing response are very similar between horses and humans, the horse also serves as an important model in tendon research [4].

Tissue engineering aims to provide functional biological substitutes of native tissue for potential clinical applications. This technique primarily involves the harvesting of specific cell types from various tissue sources, and their expansion under two- or three-dimensional culture conditions prior to their use in vivo. However, one of the most challenging aspects of this approach is maintaining the cell’s native differentiation state for long periods in culture. This is most evident in cells cultured using conventional two-dimensional systems, where differences in cell–cell and cell–extracellular matrix (ECM) contact is most evident [17]. Determining the fate of cultured cells is most readily achieved by analysing the expression of specific genetic markers. However, in the case of stem cell-derived tendon cells, this has been hampered by the lack of tendon-specific markers [7,18]. This is in contrast to other closely related tissues such as cartilage and bone, where specific molecular markers for chondrogenic and osteogenic differentiation pathways have been identified [19]. Currently, the majority of tendon-related research studies rely on the use of molecular markers such as Tenascin-C (*TNC*), Tenomodulin (*TNMD*), Scleraxis (*SCX*) and genes encoding collagen type I (*COL1A1* and *COL1A2*) as a means by which to discriminate between tenocytes and other cell lineages. However, several reports now exist, which bring into question the validity of such genes as markers of tendon tissue [19,20,21,22]. In order to help better identify more specific tendon markers, gene expression profiling techniques such as microarray analysis have been used, and identified *TNMD* and thrombospondin 4 (*THBS4*) as possible tendon-selective gene markers [23]. Recent developments in next generation sequencing now offer the possibility to profile the entire transcriptome in a very high-throughput, accurate and quantitative manner [24]. This technique has been used in equine musculoskeletal tissue to determine transcriptomic signatures associated with ageing in cartilage [25]. More recently, Peffers et al. described the use of RNA-seq in tendon for the purpose of studying differentially expressed genes in young versus old macroscopically normal human Achilles tendons [26]. We hypothesize that it is possible to identify specific genetic markers of horse tendon tissue using deep-sequencing technology. To this end, we compared gene expression patterns in various equine musculoskeletal tissues using genome-wide RNA-seq. Molecular signatures of cultured cells were also considered and compared to the corresponding native tissue.

## 2. Materials and Methods

### 2.1. Animals

Tissues samples were harvested from a total of six horses (Table 1). All horses were free from any previous history of orthopaedic disease and were slaughtered for reasons unrelated to this study. All tissue specimens used in this study were obtained following owner informed consent and were harvested in accordance with institutional guidelines and the Swiss animal protection law.

### 2.2. Sample Collection and Processing

The following tissue samples were collected under aseptic conditions immediately after euthanasia or slaughter: tendon from the forelimb region of the SDFT; ligament from the collateral ligaments of the metacarpophalangeal (MCP) joint; cancellous bone from the distal metaphysis of the third metacarpal bone; cartilage from the articular surfaces of the MCP joint; and cervical dermal tissue. Portions of the harvested tissue were then placed in RNAlater (Qiagen, Hombrechtikon, Switzerland) and stored at −80 °C, or enzymatically digested for the purpose of isolating tissue specific cells.

### 2.3. Cell Isolation and Culture

*Tendon:* Following the removal of the peritenon, tendon sections were cut into 2 mm^3^ pieces and subjected to enzymatic digestion with 0.2% collagenase I and 0.3% Dispase II (Roche Diagnostics, Rotkreuz, Switzerland) on an orbital shaker at 37 °C overnight [27]. The isolated tenocytes were cultured in flasks with Dulbecco’s modified eagle medium (DMEM) supplemented with 10% fetal bovine serum (FBS) and 1% penicillin/streptomycin (all from Thermo Fisher Scientific, Reinach, Switzerland).

*Bone*: Pieces of cancellous bone were harvested with a curette and subjected to enzymatic digestion with 0.1% collagenase II and 0.2% trypsin (BioConcept, Allschwil, Switzerland) at 37 °C on an orbital shaker for 1.5 h. The digested bony pieces were placed in a Petri dish containing bone culture medium consisting of DMEM/F-12 medium supplemented with 10% FBS, 1% penicillin/streptomycin, 0.2% Amphotericin B (Thermo Fisher Scientific) and 0.5 μg/mL ascorbic acid (Sigma Aldrich, Buchs, Switzerland) [28]. Isolated cells were transferred to culture flasks containing the bone culture medium as described above.

*Cartilage*: Cartilage samples were cut into 2 mm^3^ pieces and subjected to enzymatic digestion using 0.1% collagenase II and 0.2% trypsin at 37 °C for 3 h. Isolated chondrocytes were then seeded at a density of 1 × 10^5^ cells/cm^2^ in cell culture flasks containing DMEM/F-12 culture medium supplemented with 10% FBS and 1% penicillin/streptomycin.

*Dermis:* For the isolation of dermal fibroblasts, skin samples were collected and the subcutaneous fat layer separated. Skin was cut into small fragments of approximately 1 cm^2^ using sterile forceps and scissors. The tissue fragments were transferred to a 10 cm tissue culture dish and fibroblasts were allowed to migrate out for up to a week in DMEM supplemented with 10% FBS and 1% penicillin/streptomycin. Cells were collected when 80%–90% confluent.

All cell cultures were incubated at 37 °C, 5% CO_2_, under normoxic conditions and passaged twice upon reaching 90% confluency.

### 2.4. RNA Isolation

Cells were lysed in RLT buffer (Qiagen) and homogenized using a QIAshredder (Qiagen). Frozen tissue samples were mechanically crushed under liquid nitrogen, and homogenates were treated with RTL Buffer (tendon, ligament and cartilage) or TRIzol (bone fragments). RNA was then extracted from all samples using an RNeasy Mini Kit (Qiagen) and quality was determined using the Agilent 2200 Tape station system and the Agilent Bioanalyzer 2100 (Agilent, Waldbronn, Germany). RNA Integrity Number (RIN) values for cultured cells and tissue samples were 9.1–9.4 and 6.8–8.1, respectively.

### 2.5. Illumina RNA Sequencing and Data Analysis

Library preparation: The quality of the isolated RNA was determined using a Qubit^®^ (1.0) Fluorometer (Life Technologies, Carlasbad, CA, USA) and a Bioanalyzer 2100 (Agilent, Waldbronn, Germany). Only those samples with a 260/280 nm ratio between 1.8 and 2.1 and a 28S/18S ratio within 1.5 and 2 were further processed. The TruSeq RNA Sample Prep Kit v2 (Illumina, Inc., San Diego, CA, USA) was used in the subsequent steps. Briefly, total RNA samples (100–1000 ng) were ribo-depleted using Ribo Zero Gold (Epicentre^®^, Madison, WI, USA) and then fragmented. The fragmented samples were reverse transcribed to cDNA, end-repaired and polyadenylated before ligation of TruSeq adapters containing the index for multiplexing. Fragments containing TruSeq adapters on both ends were selectively enriched by PCR. The quality and quantity of the enriched libraries were validated using Qubit^®^ (1.0) Fluorometer and the Caliper GX LabChip^®^ GX (Caliper Life Sciences, Inc., Hopkinton, MA, USA). The product is a smear with an average fragment size of approximately 260 bp. The libraries were normalized to 10 nM in Tris-Cl 10 mM, pH 8.5 with 0.1% Tween 20.

Cluster Generation and Sequencing: The TruSeq PE Cluster Kit v4-cBot-HS or TruSeq SR Cluster Kit v4-cBot-HS (Illumina, Inc.) was used for cluster generation using 10 pM of pooled normalized libraries on the cBOT. Sequencing was performed on the Illumina HiSeq 2500 paired end at 2 × 10^1^ bp or single end 100 bp using the TruSeq sequencing by synthesis (SBS) Kit v4-HS (Illumina, Inc.). Original data is available at BioProject (www.ncbi.nlm.nih.gov/bioproject/) Accession Nr. PRJNA343028/SRP091965. Data Analysis: The raw reads were first cleaned by removing adapter sequences, trimming low quality ends, and filtering reads with low quality (phred quality < 20) using Trimmomatic [29]. Sequence alignment of the resulting high-quality reads to the Equus Caballus reference genome (Ensemble v74) was performed with tophat (version 2.0.14) and gene-level counting with HTSeq (version 0.6.1). Sequencing reads have been scanned with fastqc software and did not show quality deviations that would prohibit further analysis. To detect differentially expressed genes, we applied a count based negative binomial model implemented in the software package DESeq [30,31]. Genes showing altered expression with adjusted (Benjamini and Hochberg method) *p*-value < 0.05 were considered as differentially expressed (Supplemental Table S1). Process and pathway analysis were performed with Metacore database via GeneGo tool (Thomson Reuters, http://portal.genego.com) [32]. The pathways (groups of genes belonging to the same pathway map in GeneGo Metacore database: https://portal.genego.com/) were selected on the basis of the relevance to various biological processes (Treshold = 2; *p*-Treshold = 0.05).

### 2.6. Validation of RNA-seq

Candidate genes identified by RNA-seq were further validated using RT-qPCR. RNA (0.5 μg) was treated with Turbo DNase (Thermo Fisher Scientific) and reverse transcribed using Oligo-dT primers and Superscript III (Thermo Fisher Scientific). Real-time PCR was performed in triplicates with SYBR green (Qiagen) using a Rotor-Gene Q RG-6000 (Qiagen). Data was normalized to 18S and presented as 2^−Δ^*C*_T_. Primer design was performed using the NCBI primer designing tool (http://www.ncbi.nlm.nih.gov/tools/primer-blast/) on the Equus caballus (taxid:9796) genome. Primer pairs were selected based on the presence of at least one intron on the corresponding genomic DNA. Oligonucleotide primers used in qPCR are listed in Supplementary Table S2.

### 2.7. Equine Tenocyte Microtissues

In order to generate equine tenocyte microtissues, cells were adjusted to 2 × 10^5^ cells/mL in normal growth medium and transferred to a reagent reservoir. A total of 25 μL cell suspension was then transferred to individual wells of a Terasaki plate (VWR International, Dietikon, Switzerland) using a multistep pipette. When all wells had been filled, lids were mounted and plates inverted in order to allow for gravity-enforced microtissue formation as previously described [27,33]. After 6 days of culture, microtissue pellets were harvested and either fixed in 4% paraformaldehyde and processed for paraffin embedding, or lysed in RTL Buffer (Qiagen) and processed for gene expression analysis as described above.

### 2.8. Immunohistochemical Staining

Immunohistochemical analysis of EYA2 and chondrolectin (CHODL) was performed on rehydrated paraffin wax tissues sections (5 μm) taken from equine tenocyte microtissues and native equine tendon. Antigen retrieval was performed on native tendon tissue by incubating sections at 98 °C in citrate buffer (pH 6.0) for 15 min. Endogenous biotin and peroxidase activity were controlled for using avidin and biotin (Abcam, Cambridge, UK) and 3% hydrogen peroxide, respectively. Sections were then washed and blocked with 10% normal swine serum for 30 min and incubated with polyclonal rabbit anti-EYA2 (1 μg/mL; Abcam, UK) or polyclonal rabbit anti-CHODL (2.5 μg/mL; Biorbyt, Cambridge, UK) overnight at 4 °C or for 1 h at 37 °C. Tissue sections were also incubated with equivalent concentrations of a rabbit IgG antibody (Peprotech, London, UK) to control for non-specific staining. Sections were then washed in PBS and incubated with biotinylated swine anti-rabbit IgG (1:400; DAKO, Baar, Switzerland) for 1 h at room temperature followed by washing and further incubation for 30 min with Vectastain (Reactolab SA, Servion, Switzerland). Sections were then developed using 3,3′-diaminobenzidine tetrahydrochloride (DAB), counterstained with Harris’s Hematoxylin and mounted in Mowiol.

### 2.9. Statistical Analysis

One-way analysis of variance (ANOVA) followed by Tukey’s post-hoc test was performed for multiple group comparisons using SPSS19.0 (SPSS Inc., Chicago, IL, USA). In all cases, a *p*-value of <0.05 was considered statistically significant.

## 3. Results

Tissue engineering aims to provide functional biological substitutes of native tissue for potential clinical applications. Monolayers of adherent cells grown on flat and rigid two-dimensional substrates represent the conventional cell culture system, but one of the most challenging aspects of this approach is the incapacity of in vitro cultured cells to emulate the in vivo conditions. We therefore aimed to identify specific tendon markers, which could be used to discriminate between cartilage, bone and ligament, and help monitor the differentiation status of cultured tendon cells.

**Figure 1:**
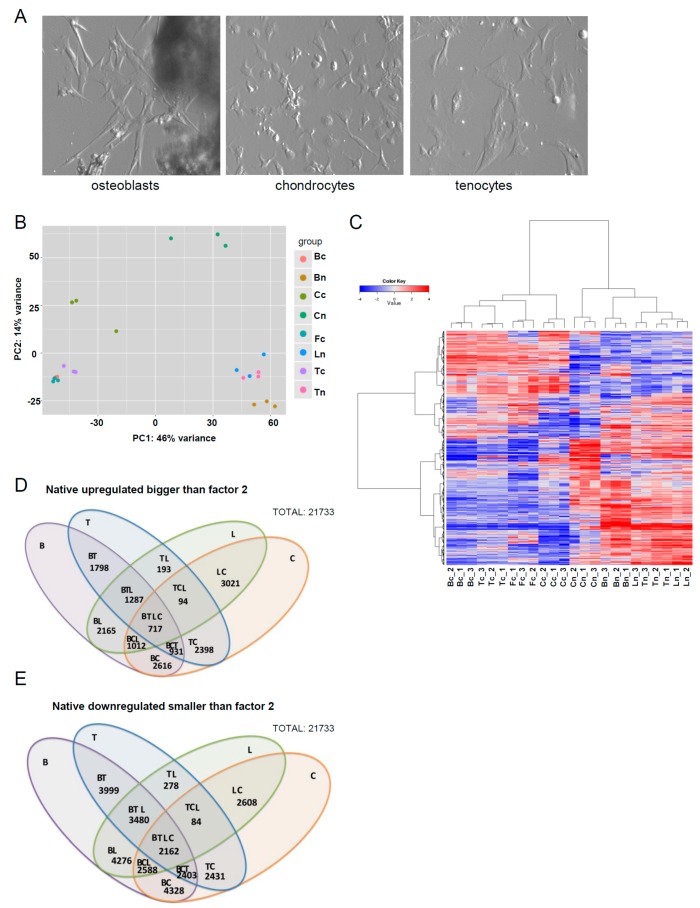
(**A**) Representative phase contrast micrographs of cultured osteoblasts, chondrocytes and tenocytes used for RNA Seq analysis; (**B**) Principal component analysis plot confirming a good separation between most of the tissue types; (**C**) Unsupervised clustering of the top 2000 genes with biggest variance across the samples; (**D**,**E**) Venn-Diagrams depicting the number of genes up- and down-regulated between all possible comparisons in native tissues. Bc, cultured bone cells; Tc, cultured tendon cells; Fc, cultured fibroblasts; Cc, cultured cartilage cells; Bn, native bone; Tn, native tendon; Ln, native ligament. 1, 2 and 3 refer to the codes of the different horses (n = 3) used in the study.

Bone, cartilage, ligament and tendon tissue were harvested from young adult Warmblood horses and in the case of tendon, tissue quality was confirmed by macroscopic and histological evaluation [34] (Supplementary Figure S1). Immediately following their collection, tissues were either directly used for RNA isolation or for the isolation of osteoblasts, chondrocytes, or tenocytes. Monolayer cultures were generated from cells extracted from tissues either by spontaneous outgrowth (e.g., osteoblasts), or by enzymatic digestion (e.g., chondrocytes, tenocytes) (Figure 1A), and RNA isolated for use in RNA-seq analysis once cells reached confluency. Dermal fibroblasts were also included as a representative non-musculoskeletal tissue-derived mesenchymal cell. A transcriptomic analysis was performed by comparing native and in vitro expanded cells. Principal component analysis confirmed a good separation between most of the tissue types (Figure 1B). Hierarchical clustering of the top 2000 genes demonstrated an obvious divergence in the clustering of RNA-seq data profiles between cultured cells and their tissue of origin (Figure 1C). The gene-level summaries have been added in the supplementary data (Supplementary Table S1), and the data is available at BioProject (www.ncbi.nlm.nih.gov/bioproject/; Accession Nr. PRJNA343028). A total of 21,733 from more than 28,000 genes tested were expressed in at least one sample. Due to the fact that a significant proportion of genes continued to pass the adjusted *p*-value threshold of 5% (also meaning 5% of false positives estimate) upon statistical filtering, we elected to define the main condition as the fold change (logFC > 2 corresponding to FC = 4). An overview of the number of differentially upregulated and downregulated genes between tissues is depicted in Figure 1D,E.

**Figure 2:**
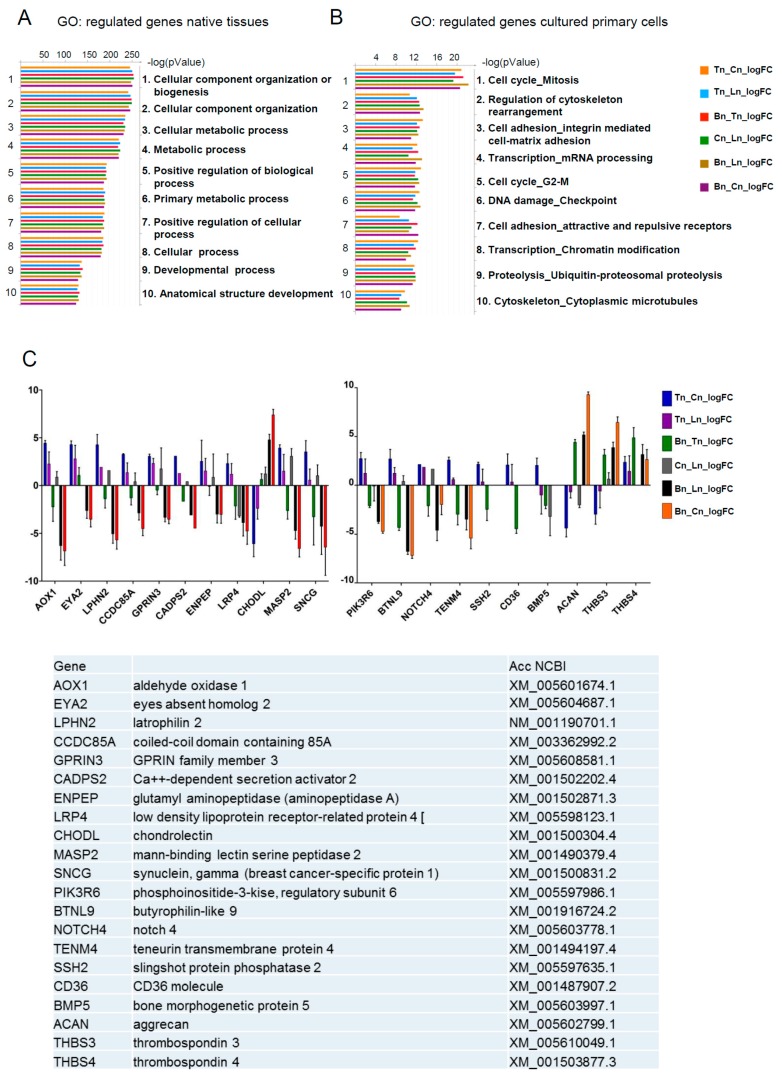
(**A**,**B**) Top ten biological process gene ontology terms as determined using GeneGo. Genes regulated in native tissues (**A**) and cultured primary cells (**B**). (**C**) Comparison of changes in expression of 21 selected genes (logFC) between native tendon (Tn), bone (Bn), ligament (Ln) and cartilage (Cn).

To gain additional insights, we performed functional annotation clustering of differentially expressed genes by Gene Ontology (GO) using the GeneGO tool. This tool allows analyses to be performed based on the translation of horse genes (which are still not completely annotated) to their mouse homologs. The pathway annotations in GeneGo included three times fewer genes (~4500) than the Gene Ontology annotations (~15,000) thus, increasing the reliability of the Gene Ontology, especially in the case of homolog translation between species. The main processes identified in native tissues samples were cellular component organization and metabolism (Figure 2A), whereas in cultured cells, the cell cycle related processes, cell adhesion and transcription were most prominent (Figure 2B). These data demonstrate that the cultivation of primary cells (tenocytes, osteoblasts, and chondrocytes) under the in vitro conditions used in the current report, leads to a substantial shift in their expected gene expression signature, and are suggestive of functional differences between native and cultured cell populations. Further analyses were therefore performed using native tissues only, and primarily focused on identifying tendon specific markers.

**Figure 3:**
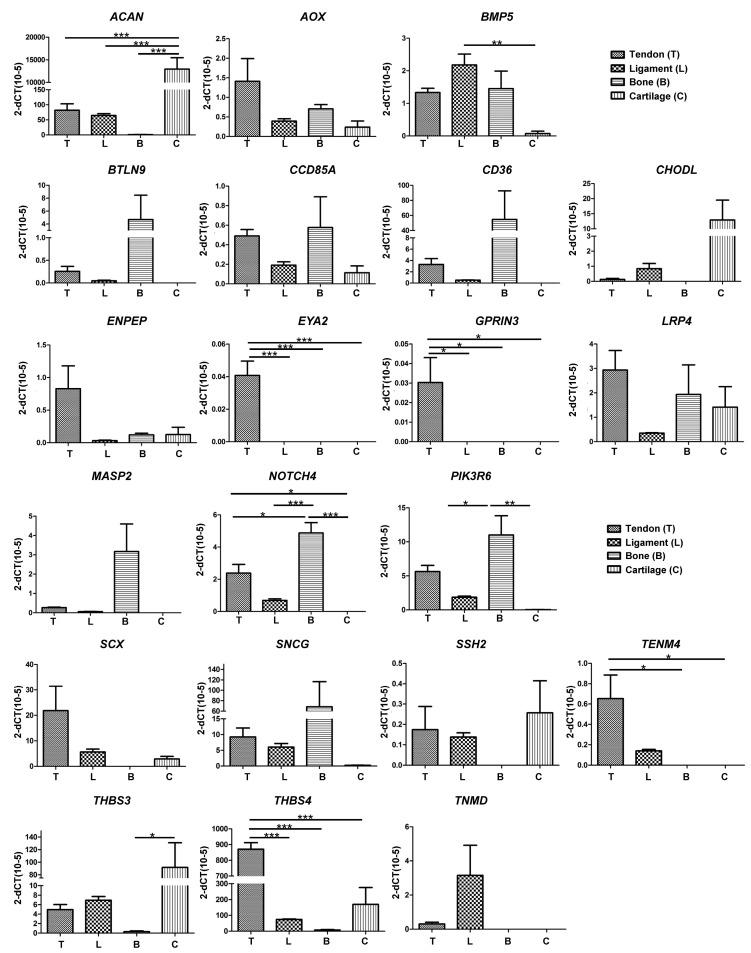
RT-qPCR analysis of candidate genes identified using RNA-seq. Data were normalized to 18S and presented as 2^−Δ^*C*_T_. * *p* < 0.01 as determined using one-way ANOVA and Tukey post-hoc test. Data are representative of three separate reactions performed using RNA isolated from three horses.

Pairwise comparisons made between the different tissue samples identified 21 candidate genes that were either over-represented or under-represented in tendon tissue as compared to ligament, bone or cartilage (Figure 2C). In order to further investigate the specificity of these genes with regards to their expression in tendon, RT-qPCR was performed on RNA from a more diverse group of horses. We identified 12 genes that were selectively expressed in either tendon (*THBS4*, *TENM4*, *SCX*, *ENPEP*), ligament (*TNMD*), bone (*BTLN9*, *CD36*, *MASP2*, *SNCG*), or cartilage (*CHODL, ACAN, THBS3*), and two genes that were exclusively expressed in tendon (*EYA2* and *GPRIN3*) (Figure 3). These data therefore confirm *EYA2* and *GPRIN3* as specific markers of equine tendon as compared to ligament, bone and cartilage.

**Figure 4:**
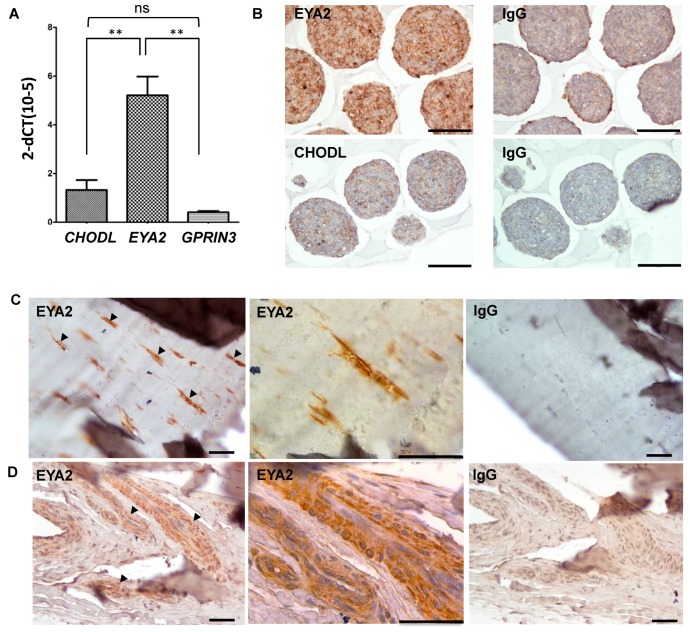
(**A**) Expression levels of *EYA2*, *CHODL* and *GPRIN3* in equine tenocyte microtissue was determined using RT-qPCR. Data were normalized to 18S and presented as 2^−Δ^*C*_T_* *p* < 0.01 as determined using one-way ANOVA and Tukey post-hoc test. Data are representative of three separate reactions; (**B**) Immunohistochemical analysis of EYA2 and CHODL in equine tenocyte microtissues. Paraffin wax sections of equine tenocyte microtissues were incubated with an anti-EYA2 antibody (**top** panel) or anti-CHODL antibody (**lower** panel) and positive staining identified using an appropriate HRP-labelled polyclonal antibody with subsequent development using 3,3′-diaminobenzidine (brown). Specificity was confirmed using the relevant IgG controls. Scale bar = 100 µm; (**C**,**D**) Immunohistochemical analysis of EYA2 in equine native tendon. Paraffin wax sections of equine tendon tissue were incubated with an anti-EYA2 antibody and positive staining was identified using an appropriate HRP-labelled polyclonal antibody with subsequent development using 3,3′-diaminobenzidine (brown). C, Tendinocytes; D Endotendineum. Specificity was confirmed using a relevant IgG control. Scale bar in all panels = 50 µm.

In order to further validate the use of *EYA2* and *GPRIN3* as markers of equine tendon, we also examined their expression levels in equine tenocytes cultured as three dimensional microtissue spheroids—an in vitro system known to help maintain the tenocyte phenotype [27]. *EYA2* expression levels were significantly increased in equine tenocyte microtissue spheroids as compared to *GPRIN3* (*p* = 0.001) and the selective cartilage marker *CHODL* (*p* = 0.004) (Figure 4A). These observations were also confirmed at the protein level, where immunohistochemical staining for EYA2 was noticeably increased in equine tenocyte microtissues as compared to CHODL (Figure 4B). Immunohistochemical analysis of EYA2 was also performed in native equine tendons, and demonstrated positive EYA2 staining in fibroblast-like cells within the tendon tissue matrix (Figure 4C) and cells localized to the vascular endothelium (Figure 4D).

## 4. Discussion

To date, only one study has compared gene expression of equine tendon with that of bone and cartilage [19]. Although significant increases in *SCX* were evident in tendon as compared to bone, no significant differences were observed in comparison to cartilage. Furthermore, *TNMD* was expressed at comparable levels in tendon and bone. Therefore, the standard markers currently used to select for tendon may not be an appropriate choice for use in horses. In order to address this issue, we used RNA-seq and RT-qPCR to compare gene expression in equine tendon, bone, cartilage and ligament tissues, and to further identify changes in the genotype of tissue-specific cells in culture.

RNA-seq demonstrated clustering of characteristic gene expression patterns according to the types of tissues, and good reproducibility between biological replicates. However, noticeable differences were observed in gene expression patterns between cultured cells and their respective tissue of origin. One possible explanation for this might be that the cultured cells underwent dedifferentiation, resulting in loss of the native genotype. Certainly, culturing primary cells as monolayers promotes dedifferentiation and is dependent on passage number [35]. Therefore, although we used cells at a low passage number, they were cultured as monolayers, which most likely influenced their gene expression pattern as compared to native tissues. Of note is that gene expression patterns in cultured cells from tendon, bone and cartilage were comparable to those observed in dermal fibroblasts. Future studies using primary cells cultured under three-dimensional (3D) conditions might provide further insights into how dedifferentiation influences gene expression [27].

RNA-seq analysis of tendon, bone, cartilage and ligament tissues revealed numerous differentially expressed gene candidates. Further validation using RT-qPCR confirmed *EYA2* and *GPRIN3* as tendon-specific genes. All the other candidate genes were expressed in more than one tissue, and therefore not considered suitable for use as specific markers for tendon. Importantly, we were unable to confirm tendon-specific expression of *TNMD*, *SCX*, or *THSB4*.

Expression of *EYA* genes, homologues of the eyes absent gene of Drosophila, has previously been described during limb tendon development [36]. Both *EYA1* and *EYA2* are widely expressed in various tissues during organogenesis and act as transcriptional activators [36]. They are also expressed early on in limb development in connective tissue precursor cells [36]. In later embryologic stages, both genes are expressed in cell condensations that form different limb tendons [36]. *GPRIN3* is a homologue of *GPRIN1*, a member of the GPRIN family of proteins involved in the downstream transduction of signals mediated via G protein coupled receptors (GPRCs) [37]. Ligands binding to GPRCs catalyse the GDP–GTP exchange of the G protein [37]. The GTP-bound form of G proteins α-subunits can then bind to various proteins, such as GPRIN1 and GPRIN2, which ultimately leads to further downstream signalling [37]. Although the specific role of GPRIN3 in tendon development and/or function remains to be determined, it is of interest to note that EYA2 can also act as a partner for G protein α-subunits [38].

Although this study is the first to identify *EYA2* and *GPRIN3* as specific markers of equine tendon as compared to bone, cartilage and ligament, previous reports have shown expression of these genes in musculoskeletal tissue from other species. The expression of both *EYA2* and *GPRIN3* has already been confirmed in human tendon tissue using RNA-seq [26]. Microarray analysis performed by Jelinsky et al. [23] has also confirmed *EYA2* expression in human and rat tendon. However, in contrast to our findings, *EYA2* was expressed in bone and cartilage [23]. The different species and methods of analysis used may help to account for these contradictory findings. Indeed, Marioni et al. have previously shown that differential expression of genes can be assessed more reliably using RNA-seq as compared to microarray analysis [39]. Even though our RNA-seq data displayed a rather high variability in gene expression between different samples—most likely as a result of the diversity within the group of horses used—*EYA2* and *GPRIN3* were consistently expressed in tendon tissue and remained absent from all other tissues tested. This therefore highlights their robustness as tendon-specific markers.

Results from our in vitro studies using cultured equine tenocytes provided further support for the use of EYA2 as a tendon marker, although failed to demonstrate any significant differences in expression levels between *GPRIN3* and the non-specific tendon marker, *CHODL*. Although we have previously identified the microtissue system as a means by which to help maintain the tenocyte phenotype in vitro [27], our findings also demonstrated that the spindle-like morphology typically associated with mature tendon cells was only fully realized when microtissues were embedded in collagen gels. Gene expression analysis in tenocyte microtissues cultured under conditions more representative of native tendon may therefore be warranted. In addition to *EYA2* gene expression, we were also able to confirm EYA2 protein expression in equine tendon tissue. Immunohistochemical staining of paraffin-embedded sections demonstrated positive staining for EYA2 in tenocytes cultured under 3D cultures, and in elongated fibroblast-like tendon cells in native tendon tissue. Blood vessels within tendon tissue sections also stained positively for EYA2. Due to anatomical and cellular heterogeneity of tendon [40,41], additional studies will be required to determine the spatial and temporal expression of EYA2 in equine tendon. Finally, although we have presented evidence of *EYA2* and *GPRIN3* as specific markers of equine tendon in comparison to bone, cartilage and ligament, investigations are now needed to assess their expression in other species. Taken together, our data suggest that the combined use of the newly identified tendon-specific markers *EYA2* and *GPRIN3* with other more well established selective markers such as *SCX* and *TNMD*, could be an effective means by which to unambiguously determine the identity of tendon tissues generated in vitro.

## 5. Conclusions

In conclusion, we have established *EYA2* and *GPRIN3* as two specific molecular markers of equine tendon tissue in comparison to bone, cartilage and ligament. Our in vitro data also support the use of EYA2 as an additional means by which to examine the differentiation status of cultured tenocytes. Finally, based on the observed differences in gene expression between cultured cells and their tissue of origin, we surmise that the way in which ex vivo material is processed and maintained in culture may have important implications for tissue engineering applications.

## Figures and Tables

**Table 1:** Details of horses used in study.

Code	Breed	Sex	Age (years)	Studies Performed		
				Cell Isolation	RNA-seq	RT-qPCR
1	Warmblood	Mare	6	✓	✓	
2	Warmblood	Gelding	4	✓	✓	
3	Warmblood	Gelding	3	✓	✓	
4	Lipizzaner	Gelding	11			✓
5	Warmblood	Mare	15			✓
6	Warmblood	Mare	5			✓

## Supplementary Materials

The following are available online at www.mdpi.com/2073-4425/7/11/97/s1, Figure S1: Representative pictures of haematoxylin and eosin stained tendon tissue sections from the three horses used for RNA-seq experiments confirming good quality, non-degenerated tissue, Table S1: Analysis of RNA Seq Data, Table S2: Table showing oligonucleotides used in qPCR reactions.
