# Supplementary material for: Identification of Novel Equine (Equus caballus) Tendon Markers Using RNA Sequencing
Source: Genes (Basel). 2016 Nov 10;7(11):97. doi: 10.3390/genes7110097 (PMC5126783; doi:10.3390/genes7110097)
Supplement: Supplementary file 1 [file genes-07-00097-s001.zip › genes-07-00097-s001/genes-127145-Supplementary Figure S1 and Table S2.docx]

Supplementary Materials: Identification of Novel Equine (*Equus caballus*) Tendon Markers Using RNA Sequencing

Jan M. Kuemmerle, Felix Theiss, Michal J. Okoniewski, Fabienne A. Weber, Sonja Hemmi,
Ali Mirsaidi, Peter J. Richards and Paolo Cinelli


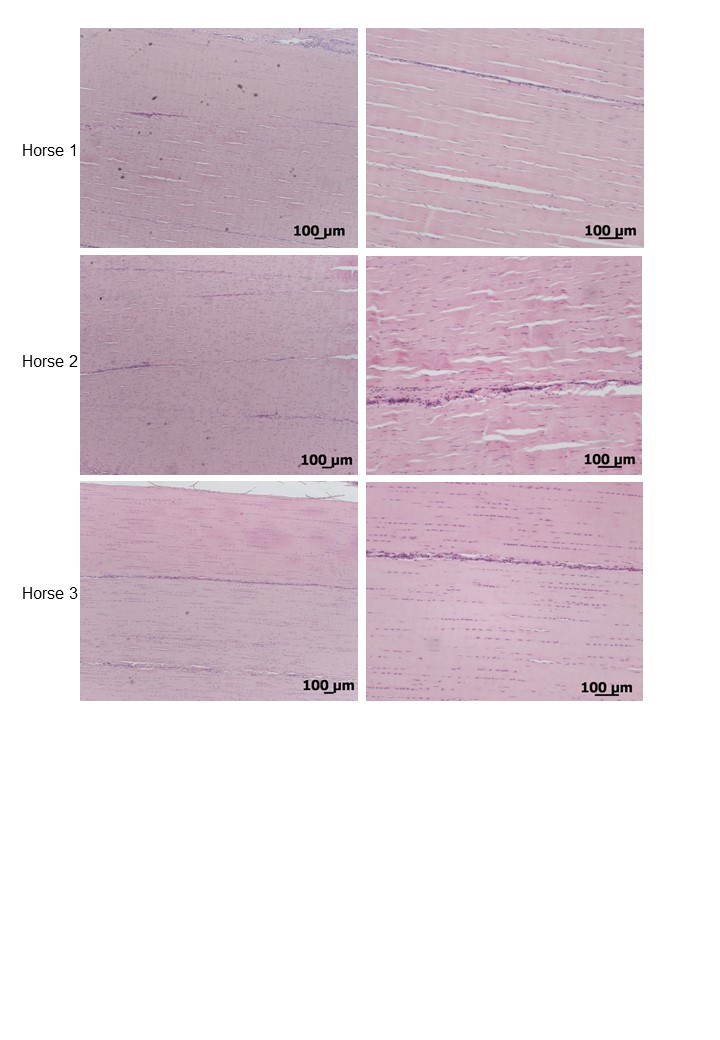


**Figure S1**. Representative pictures of haematoxylin and eosin stained tendon tissue sections from the three horses used for RNA-seq experiments confirming good quality, non-degenerated tissue.

**Supplementary Table S2.** Table showing oligonucleotides used in qPCR reactions.

|  | FWD Primer (5'-3') | BWD Primer (5'-3') |
| --- | --- | --- |
| AOX1 | GGAAATAGACTGCCTGACAGAACA | CCACGAGTGTACAGAACTCCC |
| EYA2 | AGCACGTACAACTTCTCCGC | GGCATGTTGTGCTTTTTCGC |
| LPHN2 | CCCCTTTCCCAACCAGTACC | CGCAGAATCTCTCTTGGGCA |
| CCDC85A | CATGCTGCCCCAGGTTGTATG | GGCAGTGGTGATCTCATGTTTG |
| GPRIN3 | AGCTTAGACTCTGAACATATGAGG | CCAGGGTCTACGTATGCAGG |
| CADPS2 | GCTCAGCTTTATGCAGACCG | GAGAGGCGCAGAATGCAAAG |
| ENPEP | AGAGTGGCAGATGCTTGACC | TAGCCCATCTGTCTCGTCCA |
| LRP4 | CTCACTCAGCAGGACAGGTG | ACAGGCACACACAAAGTCCA |
| CHODL | GCGGAGAGAGTAGGGTCAGA | AAACACACCTTTTGGCCGCT |
| MASP2 | CAGCAGAGGACATCGACGAG | TCTTGAGGGAGTCGTAGGGG |
| SNCG | AGAGAAACACCCCTGCCTTG | CAGCAGCATCTGTGGGTCAT |
| PIK3R6 | GGGACGTCGTCAGATTCGAG | ACTCACCGGCTCCTAGACAT |
| BTNL9 | AGAGAGCTTTCCTCGGGACT | CCTTCTCCAGTCAAGCTCCG |
| NOTCH4 | AGCCAAGGCACAGAAGTCTC | ACAGGGCTGGGATTGACAAG |
| TENM4 | CCGGATCCTGTATGACCACG | GCAGCACCATGGACTTCTCT |
| SSH2 | GTCCAGCGGTCGCCTAC | AAGCTCTCACTGATGCTCCT |
| CD36 | AGGATTCAGTGCAGGGCTTTT | TTCCAAGGGGTCTGGTTCTTC |
| BMP5 | ACCGGAGCAACAGTCGATTT | ACTGCGTCCATCTCCTGTTT |
| ACAN | GCGGTACGAGATCAACTCCC | GCGACAAGAAGAGGACACCA |
| THBS3 | TTGAACGCAATGGTGCAGTG | GGCAGTTGTCCTGTTTGCAG |
| THBS4 | CACGTAAACACCCAGACGGA | TCGTACCCTGATGTAGCCCA |
| TNMD | TCCCACTCTAATAGCAGTTTCAGA | TTGCCTCGACGGCAGTAAAT |
